# Effects of co-players' identity and reputation in the public goods game

**DOI:** 10.1038/s41598-023-40730-4

**Published:** 2023-08-19

**Authors:** Waldir M. Sampaio, Ana Luísa Freitas, Gabriel G. Rêgo, Leticia Y. N. Morello, Paulo S. Boggio

**Affiliations:** 1 Social and Cognitive Neuroscience Laboratory, Mackenzie Presbyterian University, Rua Piauí, 181, 10Th Floor, São Paulo, 01241-001 Brazil; 2 National Institute of Science and Technology on Social and Affective Neuroscience, São Paulo, Brazil

**Keywords:** Psychology, Human behaviour

## Abstract

Players’ identity and their reputation are known to influence cooperation in economic games, but little is known about how they interact. Our study aimed to understand how presenting pre-programmed co-players’ identities (face photos; names) along with their previous cooperation history (reputation) could influence participants’ cooperative decisions in a public goods game. Participants (*N* = 759) were allocated to one of six experimental groups: (i) control (no information); (ii) only reputation (neutral, free-rider, or cooperative); (iii) only face; (iv) face with reputation; (v) only name; (vi) name with reputation. In the reputation group, cooperation significantly decreased when free-riders were playing and significantly increased when they were cooperators. Person’s identity affected cooperativeness only when combined with reputation: face photo mitigated the negative effect of the free-rider reputation, while name identity mitigated any significant effect expected for reputation. Our study suggests a hierarchy: reputation changes cooperation, but a person's identity can modulate reputation.

## Introduction

Cooperation is crucial for group maintenance and survival^1^. It occurs when two or more people in an interdependent relationship act together to reach mutually beneficial outcomes^2^. It has been relevant in behavioral and social sciences studies since understanding what makes people cooperate can solve problems associated with individual overconsumption of public resources, the “tragedy of the commons” problem^3^.

A well-known method to assess cooperation is through games, like the public goods games (PGG)^4^. In the PGG, three or more players receive some initial tokens and decide to contribute any amount to a public fund that will multiply all donations and equally share the outcome among all players. However, the players have a strong incentive to free-ride since defective players will always win more than cooperators—if everyone invariably receives the share, a player will have better outcomes if free-riding when others cooperate^5^.

PGG studies have shown that cooperation is context-dependent since contextual variations may modulate cooperative decisions given differences in benefits, costs, or risks^6^. For example, in PGG experiments with anonymous players cooperation rapidly decreases^7^. However, some studies show that unmasking players through (i) identity revealing, by displaying face photos (e.g., Refs.^8,9^); or (ii) reputation informing, by displaying the previous history of a player’s behavior (e.g., Refs.^7,10^), influences cooperative behavior, since knowing who we are playing with allows evaluations of the chances of reciprocity and modulates our expectations, once we know how our co-players acted before^7,8^.

Regarding a person’s identity, studies indicate that both showing the participant's face photo to other players (e.g., Ref.^11^), or showing other players’ faces to the participant (e.g., Ref^8^) increases cooperation. Specifically, seeing others’ faces in social interactions seems to enhance not only cooperation but prosocial behaviors in general^12,13^. Diverse hypotheses explain this effect, like automatic processes associated with the sensation of being observed^14–16^, or the influence of facial traits^17^, to more complex aspects since faces are a relevant feature for social identification, allowing the recognition of cooperative or defective players in future encounters^10,18^. It is also possible that face pictures have an effect of increasing humanization, therefore, facilitating cohesion and cooperative behavior^19^.

Reputational information of other players (i.e., their cooperation decision in previous turns) is a crucial factor in modulating cooperative decision-making^20^, because it allows the decider to infer the risk of another player defecting and it is also associated with group mechanisms that are relevant to social cohesion, such as reciprocity^7,21^ or punishment of non-cooperators^22^. For instance, in a PGG study, the reputation of group leaders enhanced the investors’ cooperation^23^.

Despite several studies investigating reputation and a few others assessing players’ identity in PGG, to our knowledge only the study by Andreoni and Petrie^8^ investigated how both factors, alone or interacting, modulated the participants’ decision to cooperate with a potentially underpowered sample size (n = 20). In their study, the participants played a PGG in different conditions, such as seeing the 4 co-players’ face photos or the last financial contribution of each member of the large group (not knowing who exactly they were playing with), seeing only photos or information of the co-players, or no information. Their conclusion was that faces enhanced cooperation, other information had no effect, and information and photos together had a considerably greater positive effect compared to other conditions. Subsequent analyses showed that groups with more cooperative co-players’ led to more cooperative participants, while groups with less cooperative co-players led to less cooperative participants, which points to a reputation effect on cooperative behavior.

Previous research, with exception of Andreoni and Petrie’s^8^, have extensively explored the effects of identification and reputation on cooperation in public goods games separately^7,9–14^, ignoring that these variables often co-occur in real-life social interactions. Thus, understanding how identification and reputation interact is essential for obtaining results that more accurately reflect everyday social dynamics. Our research aims to address these limitations by utilizing a larger, more robust sample and examining the impact of an additional identification variable, namely ‘names’, to determine whether the observed effects are specific to face photos or attributable to identification variables more generally.

Name and photo are pieces of information that can lead to the identification of co-players in the PGG. However, several other functions are specifically attributed to face photos, such as the sensation of being observed^15^ and the perception of facial characteristics^24^. By studying names and face photos, this study is able to control for and examine whether the effects can be generalized to any identification variables or if they represent a unique case when presenting face photos. Here, the examination of the interactions between identification and reputation variables can contribute to a more nuanced understanding of their combined influence on cooperation in public goods games.

As demonstrated, face photos seem to interact with reputation, but can this type of information modulate any type of reputational information (e.g. from selfish to altruistic)? Would the face identity effect also be similar with other types of identification (e.g., name)? To answer these questions, we designed a large-scale (n = 759) between-groups study, manipulating a PGG with two contextual variables, so we could see how they would influence group-based cooperation: (i) player’s identity, showing different types of co-players’ identification (none, face photo, name); and (ii) pre-established reputation (neutral, free-rider, cooperative), informing the participants about the co-players’ previous contributions in the last PGG rounds. In this study, we attributed a single reputation to all three co-players in the PGG for methodological reasons, as individual reputations would require numerous distinct participant-reputation sets and increase complexity, potentially causing participant inattention in an online experiment.

We hypothesized that, compared to the control group, inserting contextual variables would influence cooperative responses. Specifically, presenting just face photos should increase the probability of cooperation because it allows a positive effect of observation and humanization and enables mechanisms of reciprocity. Regarding the display of reputation indexes, we expect that a cooperative reputation increases cooperation, while a neutral reputation would not differ from the control group, and a free-rider reputation would increase free-rider responses. By associating reputation indexes with face photos, we expect that photos will enhance the said effect of the indexes because they allow reciprocity. Regarding groups with names, since names are relevant components of someone’s identity^25^, we expect similar effects to photos because they should allow the effects of reciprocity and humanization, but not observation.

## Methods

### Participants

An a priori sample size calculation was performed using the ‘pwr’ package to determine the number of participants needed, considering a desired power of 95%, a 5% alpha level, and a small effect size (f = 0.2). The result of the calculation was a total sample size of 83.3 per group. Previous research on this topic may have suggested larger expected effect sizes, but our study took a more parsimonious approach and adopted a small expected effect size in our sample size calculation. This decision was based on the unique methods and hypotheses of our study, which differed in some ways from previous research in this area. By using a small expected effect size, we were able to ensure that our study was adequately powered to detect even modest differences between groups. Considering the possibility of sample loss due to exclusion criteria, we targeted a sample of at least 120 participants per group.

**Figure 1:**
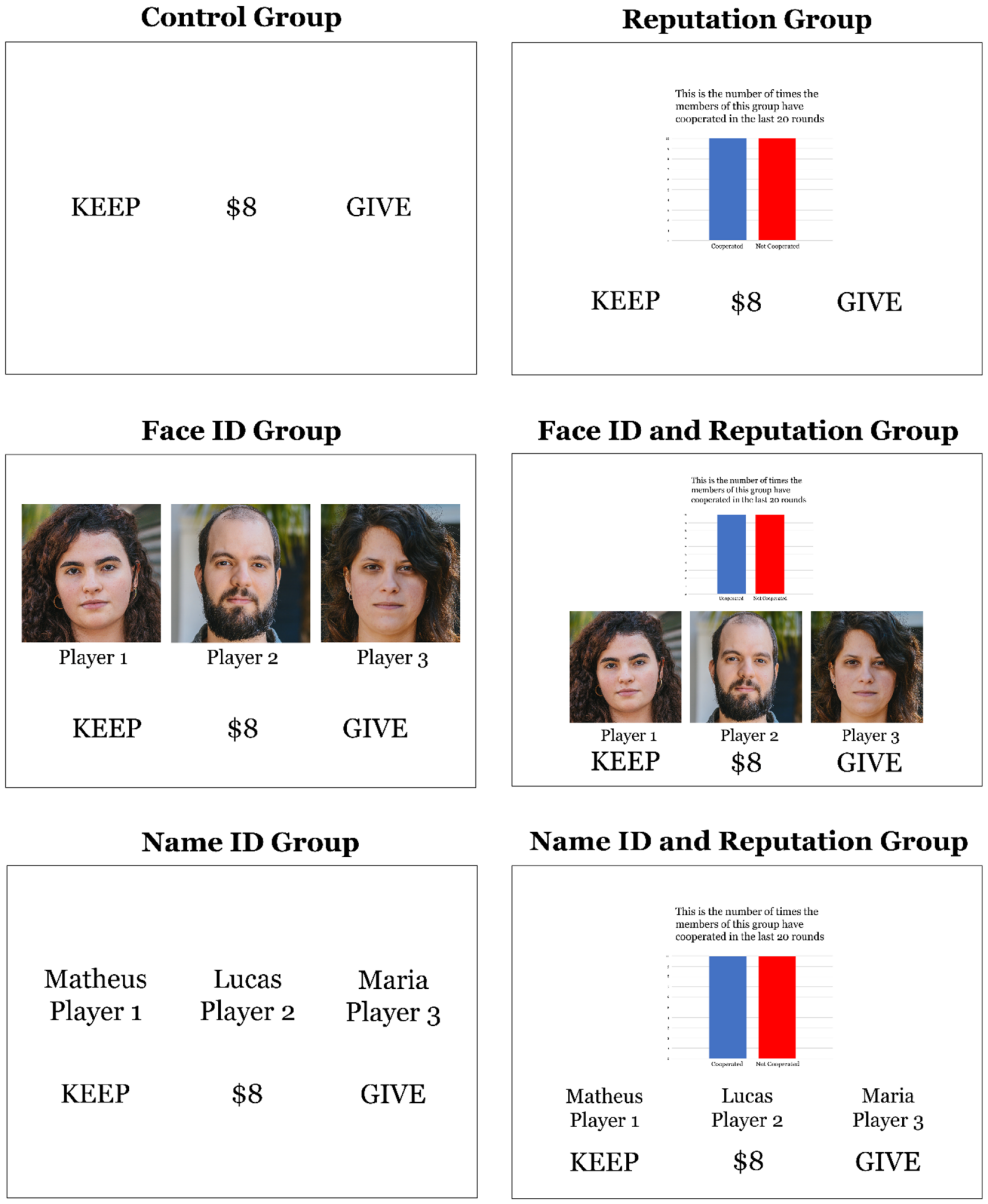
Overview of the decision screens for each experimental group. Note. Created by the authors. The face photos presented here were taken by the authors and are similar to those used in the experiment.

A total of 759 participants (M_age_ = 38.1, SD = 12.4, 51.9% female, 47.2% male) volunteered for the experiment, which consisted of playing an online version of a PGG. We included participants from 18 to 72 years old and excluded those who failed the attention check and specific PGG responses that were longer than 20 s. Participants were randomly allocated to one of the six experimental groups, which differed on the information they had from other players during the PGG: (i) control (no information regarding identity or reputation); (ii) only reputation (neutral, free-rider, or cooperative); (iii) only face; (iv) only name; (v) face and reputation; (vi) and name and reputation (see Fig. 1).

Specialized companies in data collection recruited all participants, sampled and collected in quotas by age and racial identification based on the representativeness of the Brazilian population. Offerwise (Brazil; https://www.offerwise.com/) collected the data from both groups containing the name identification, while Respondi (Germany; https://www.respondi.com/) collected the data for the other groups. To ensure data quality and prevent duplicate responses from the same participants, the companies verified the uniqueness of each account used in the study. Additionally, we implemented an attention check midway through the experiment by asking participants to write the word “ball” in a text box; those who failed this check were excluded from further analysis. Moreover, we excluded participants with response times below 200 ms or above 20 s, as these may indicate inattention. Lastly, we believe that excluding individuals who exclusively cooperated or derogated also helped eliminate participants responding automatically.

We informed all participants that they could abandon the experiment at any moment, and they all gave their informed consent. The whole experiment received approval from the Mackenzie University Institutional Ethics Committee (SISNEP, Brazil; CAAE: 46235921.0.0000.0084) and strictly followed the Declaration of Helsinki.

### Instruments

#### Public goods game

We adopted a four-player one-shot PGG version similar to that used by Hackel and colleagues^26^, adapted as an online task using the psytoolkit tool^27,28^. The co-players were all pre-programmed by the researchers. The game was preceded by an instruction screen informing the participants that they were not interacting in real-time with the other players and that their contributions shown in each round were recorded previously. We also informed the participants that their responses would be recorded for future experiments. The instructions given were as follows: “Likewise, your responses will be anonymously recorded and used in future studies”. This way, they knew their answers could be used in future studies, but not exactly in the same experiment they were participating in. The game consisted of 33 rounds and in each one the participant interacted with three unknown players, who appeared only once during the game. Participants were not informed how many rounds they would play prior to initiating the game. In each round, they received a fictitious amount of R$8.00, which they could either keep for themselves or give any amount to a “public fund”. We informed the participants that each player’s contribution would be doubled and equally divided among all four players. At the end of each round, they saw feedback screens showing the other players’ choices, the amount collected in the fund, and the amount shared from the public fund. Each trial was composed of four screens: a first screen showing players’ identities as seen in Fig. 1, where the participants should choose between keep or give; and three feedback screens, showing others players’ decision (keep or give), the total amount in the fund, and its sharing between players, respectively (each one with 3 s). It is important to highlight that the sequence of co-players' decisions was the same in every group, so any effect of order would be the same for all participants.

The six experimental groups played the same version of the PGG; however, they differed in what information the participants would have about the other players at the beginning of each round. The information could be the players’ identity (name or photo) or/and a group reputation of the other players, indicating the sum of how many times the three players cooperated in the previous 20 rounds. The Reputation Group consisted of: free-rider (≤ 25% of cooperation in the last 20 rounds); neutral (≥ 50% and < 75% of cooperation in the last 20 rounds), and cooperative (≥ 75% of cooperation in the last 20 rounds). These reputation levels were displayed as bar graphs on the screen, as illustrated in Fig. 1.

It is relevant to highlight that the other players were not real—their decisions were pre-programmed by the researcher, and the photos and names used were not from real people. Regarding other players' decisions, during the 33 rounds all the participants in the experiment were subjected to the same players' decisions, in the following proportion: 0 givers (2 rounds), 1 giver (10 rounds), 2 givers (15 rounds), 3 givers (6 rounds). Given this proportion, on average other players contributed to the public fund 59% of the time. The photos used in the experiment were generated by Generative Adversarial Networks (www.thispersondoesnotexist.com), to avoid any possibility of the people being recognized by any participant of the current study. To reduce the probability of bias towards a specific racial minority group, we decided to validate photos by people’s skin color and chose just those perceived as white. Using the software, we manipulated the pictures to present a neutral facial expression. Details about the selection, manipulation, validation, and the chosen photos are available in supplementary materials (Sect. 6). Regarding the names, we avoided names typically associated with specific socioeconomic or racial groups. We chose 99 names (3 names per round, 33 rounds in total) from the list of the 100 most registered birth names in Brazil in 2018^29^; see supplementary material Sect. 7 for details. Participants were debriefed only at the end of the experiment.

### Procedure

The participants recruited by the companies received an internet address directing them to the experiment. Firstly, they were presented with the instructions for the experiment, as well as an informed consent form explaining other details concerning the experiment, such as benefits, risks, anonymity, data storage, and the contact information of the responsible researcher. In case of agreement with the informed consent, they answered demographic questions about sex (male, female or other), age (in years), income (9 levels: R$1–R$500; R$501–R$1,000; R$1,001–R$2,000; 2,001–R$ 3,000; R$ 3,001–R$5,000; R$5,001–R$10,000; R$10,001–R$20,000; R$20,001–R$100,000; R$100,001 and above), and racial identification (white; black; asian; mixed-race (pardo); native Brazilian indigenous). The participants then played the PGG. In the end, they were debriefed about the other players.

### Statistical analysis

We recorded participants’ responses, i.e., if they cooperated with the public fund in each round (variable ‘Response’; levels: 0 = Free-ride and 1 = Cooperate), as well as the reaction time (RT) for each proposal. Based on the RT, we marked extreme responses (i.e., shorter than 200 ms or longer than 20 s) as null, since they could indicate inattention to the game. Then, using a log-transformed RT, we detected and marked outlier responses as null, using the interquartile range (IQR) “rule” (i.e., considering the range of 1.5 times the IQR beyond the first and third quantiles). After marking the null values, we excluded participants with more than 20% of null responses. Finally, we detected and excluded participants who responded equally in all the rounds (i.e., always derogating or cooperating), as they could be considered insensitive to the experiment’s manipulation. Our decision to exclude invariant responses was driven by both theoretical and methodological considerations. Our experimental stimuli, specifically the reputation indexes, would elicit variations in cooperative behaviors, thus participants whose responses remained unchanged across different contexts might not be reacting meaningfully to the stimuli. Additionally, in economic games such as the ones employed in our study, certain invariant response patterns might be considered optimal, but their exclusion is justified as our focus is on understanding the variability of participant behavior in the face of varying social cues and dynamics. It is also important to note that invariant answers may indicate that a participant may not be actively engaged in the experiment^30^, which is a point of high concern in online studies where the capacity to control a participant’s behavior is already limited.

For the main analysis, we fitted a logistic mixed model using the ‘gmler’ function from lme4 package^31^ with maximum-likelihood estimation and the ‘BOBYQA’ optimizer to predict the ‘Response’ variable (i.e., decision to cooperate in the PGG) given ‘Condition’ factor. The ‘Condition’ factor was created based on the six experimental groups, where those assessing reputation were decomposed, each one into three conditions (free-rider, neutral, and cooperative), summing a total of 12 levels, where: (0) Control (Reference group in the model); (1) only reputation—free-rider; (2) only reputation—neutral; (3) only reputation—cooperative; (4) only face; (5) only face and reputation—free-rider; (6) only face and reputation—neutral; (7) only face and reputation—cooperative; (8) only name; (9) only name and reputation—free-rider; (10) only name and reputation—neutral; (11) only name and reputation—cooperative. To control random effects of participants and experimental groups, the model included participant ID nested within the Group as random effects. The formula of the main model was ‘Response ~ Condition + (1|ID/Group)’. We compared the main model with secondary models, each one adding other predictors, which is described in detail in the supplementary materials (Sects. 1–4). We found no significant difference when these other predictors were added. Standardized parameters were obtained by fitting the model on a standardized version of the dataset. 95% Confidence Intervals (CIs) and *p*-values were computed using the Wald approximation. We ran all the analysis using R and R Studio^32^.

### Ethical approval

For the purposes of this manuscript, which includes information or images that could lead to identification of a study participant, informed consent has been obtained from all subjects to publish the information/image(s) in an online open-access publication. All procedures followed were in accordance with the ethical standards of the responsible committee on human experimentation and with the Helsinki Declaration of 1975, as revised in 2008.

## Results

All of the 759 participants completed the experiment. Regarding null responses, we excluded 29 participants (3.82%). From the remaining 730 participants, we detected and excluded 34 (4.66%) due to invariant responses, i.e., those who always derogated or cooperated. The demographic data for the 696 participants are described in Table 1, along with the averaged cooperation rate by group. We tested differences among the six experimental groups on sociodemographic characteristics, such as sex [χ^2^(5) = 4.43, *p* = 0.49] and age [*F*(5, 684) = 0.90, *p* = 0.48], which were non-significant. We also compared the number of participants in the six experimental groups across nine income brackets, and the only significant difference was detected in the bracket regarding participants receiving between R$1,000.00 and R$2,000.00 (in 2022, the Brazilian minimum wage is R$1,212.00), with more participants in this income bracket in the experimental groups “only name” and “name with reputation” compared to the others. The significant tests concerning income are presented in the analyses’ code (supplementary materials).

**Table 1:** Sample size, sex and mean age (in years), and mean cooperation rate of participants in each experimental group.

Group	n	Mean age* (SD)	Sex (n)* female/male/other	Mean cooperation % (SD)
Control	108	37.87 (12.04)	55/51/1	48.3 (21.3)
Reputation	116	39.23 (13.35)	56/57/0	48.1 (19.3)
Face ID	114	38.11 (12.86)	59/54/0	44.9 (22.2)
Face ID and reputation	114	38.58 (12.79)	61/53/0	50.3 (19.4)
Name ID	131	37.42 (12.59)	76/54/0	49.1 (22.0)
Name ID and reputation	113	36.00 (10.54)	51/61/1	46.4 (21.1)
Total	696	37.86 (12.40)	358/330/2	47.89 (20.92)

*There were missing responses for Age and Sex in the following groups: Control (1 missing); Reputation (3 missing); Face ID (1 missing); Name ID (1 missing).

### Behavior on PGG

**Figure 2:**
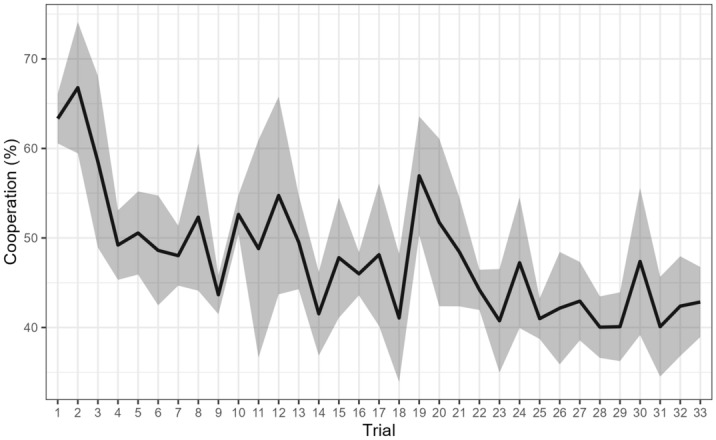
Cooperation occurrence along the trials. Note. Proportion of cooperation occurrence in each trial in all groups. Gray ribbon shows 95% CI.

Overall, participants tended not to cooperate in the PGG (cooperation rate mean: 47.89%; SD: 20.92%). Based on the cooperation rate values for each participant, we compared the cooperation rate between experimental groups through a one-way ANOVA, which indicated no main effect for Group [*F*(5,690) = 0.99, p = 0.43]. Despite most of the participants cooperated in the first round (63.36%), this number significantly decreased in the last round (42.96%; [χ^2^(1) = 27.25, *p* < 0.001]). This decreasing tendency can be seen in Fig. 2 and was present in all experimental groups, as shown in Sect. 5 (supplementary materials).

Regarding reaction time in the PGG, we ran a mixed ANOVA (Satterthwaite’s method) using averaged log-transformed RT as the dependent variable, and factors *group* (the six experimental groups) and *cooperation response* (free ride or cooperate), which showed significant main effects for both factors [*cooperate*: F(1,690) = 114.96, p < 0.001, η_*p*_^2^ = 0.14; and *group*: F(5, 948.5) = 27.25, p < 0.001, η_*p*_^2^ = 0.13], but no interaction for cooperation*group [F(5, 690) = 1.52, p = 0.18, η_*p*_^2^ = 0.01]. Regarding cooperation decisions, overall, participants were slower to cooperate (Median = 2195 ms; IQR = 2331.25 ms) in comparison to free-riding (Median = 2005 ms; IQR = 3861.81 ms). Concerning the factor *group*, post hoc analysis showed that participants in both “control” and “name and reputation” groups were significantly faster compared to all other groups, while they were not significantly different from each other. Post hoc results for the RT analysis are described in the analyses’ code (supplementary materials).

### Predicting cooperation from condition

We ran a logistic mixed model to predict Cooperation from the Condition variable, with the control group as reference. Regarding the random effects, the Group:ID variance is 0.16 (SD: 0.40), and ID variance is 0.70 (SD: 0.84). Coefficient estimates, confidence interval, and *p*-values of the model are described in Table 2.

**Table 2:** Logistic mixed model estimates and standard error (SE) for each term, with 95% confidence interval and *p*-value.

Term	Estimate	SE	95% CI	*p*-value
Intercept	−0.07	0.09	−0.26, 0.12	.46
Face ID	−0.15	0.13	−0.42, 0.11	.24
Reputation Free-Rider	−0.30	0.13	−0.58, −0.03	.02*
Reputation Neutral	0.30	0.17	−0.03, 0.65	.07
Reputation Cooperative	0.38	0.14	0.11, 0.67	.007**
Face ID and Rep. Free-rider	−0.13	0.13	−0.41, 0.14	.32
Face ID and Rep. Neutral	0.48	0.17	0.14, 0.83	.005**
Face ID and Rep. Cooperative	0.34	0.14	0.07, 0.63	.01*
Name ID	−0.09	0.13	−0.36, 0.17	.46
Name ID and Rep. Free-Rider	−0.10	0.13	−0.37, 0.16	.43
Name ID and Rep. Neutral	0.16	0.16	−0.16, 0.49	.32
Name ID and Rep. Cooperative	0.23	0.13	−0.03, 0.51	.08

***0.001; **0.01; *0.05.

**Figure 3:**
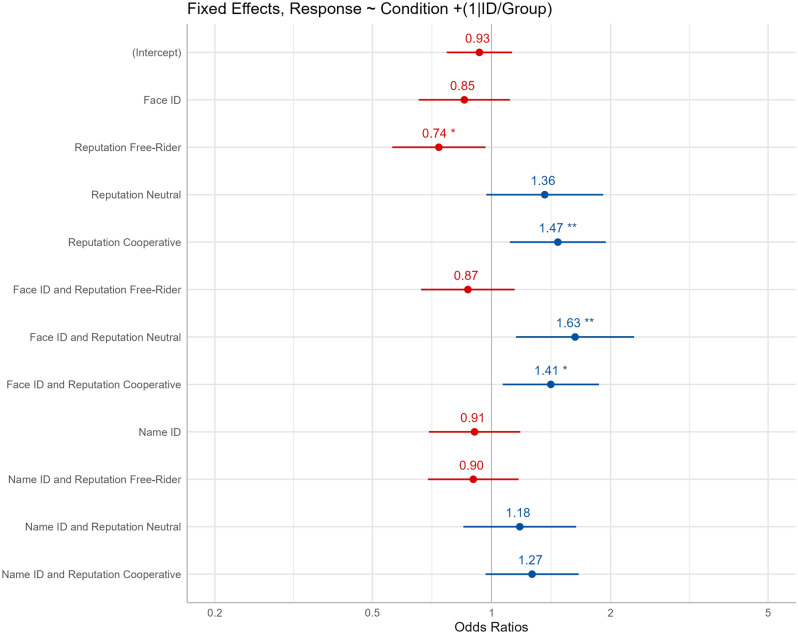
Odds ratios for the fixed effects. *Note.* Created by the authors; **p < 0.01, *p < 0.05.

Overall, the model showed that, compared to the control group, the ‘only reputation’ group significantly decreased cooperation in the free-rider condition and increased cooperation during cooperative conditions. Additionally, the ‘face and reputation group’ significantly increased cooperation during neutral and cooperative conditions. Although not significant, we found statistical tendencies for the ‘only reputation’ group in neutral conditions, and also for the “name and reputation group” in cooperative conditions. Those detected effects and their odds ratios can be seen in Fig. 3.

Finally, we evaluated secondary models by adding sociodemographic factors (sex, age, and income) to determine if they would impact the findings of our main model. None of the aforementioned sociodemographic factors had any effect on the findings from the main model.

## Discussion

In the current study, we investigated how the co-player’s identity and reputation interacted and influenced the participants’ cooperative decisions. Merely revealing co-players’ faces or names was insufficient to modulate cooperation. However, knowing the others’ reputations significantly decreased participants’ cooperation, when co-players were free-riders and increased it when others’ reputation was neutral and, when they were cooperative, cooperation was significantly positive. In groups with reputation indexes, we observed a hierarchical relationship when faces were presented alongside reputation graphs. Our findings show that reputation modulates cooperation, and faces influence the impact of reputation on it.

Previous studies found a positive modulatory effect of face identification on cooperation^8,9^, while our results show that merely displaying face photos is insufficient to generate this effect. The habituation process could explain the absence of this effect, as unchanged stimuli tend to desensitize observers’ responses over time^33^. However, we are skeptical about extending this explanation to our data because studies based on this hypothesis mostly used eyespots as cues^33^, not faces or eyes. One major strength of our results is the significantly positive effect observed when we displayed face photos with reputation graphs (neutral and cooperative). This design contained more information to process, consequently resulting in more attentional shifts and a lower probability of habituation. Most importantly, we argue that face photos alone may not positively influence cooperative decisions but are relevant to humanize^19^ who we are playing with since the presence of face photos diminished the negative effect of free-rider reputation on cooperation.

In this study, we reject the observation effect hypothesis^15^, because we have evidence leading away from this traditional explanation. We assume that the given face ID contextual factor alone is not enough to make participants infer the co-players’ behavior, but it is a stronger modulator of other contextual variables, like reputation. Knowing that manipulating expectations alters the acceptance likelihood of unfair offers^34^, we manipulated them by adding reputation indexes. In doing so, the influence of reputation on cooperation became evident, and moreover, with face photos, the reputational information was manipulated as well.

Our findings indicate that the presence of face photos can effectively mitigate the negative impact of a free-rider reputation on cooperation. In real-world group settings, such as workplace teams, community organizations, and online platforms, incorporating visual identity cues, like profile pictures, has the potential to foster a more cooperative environment, even among individuals with a history of less cooperative behavior. In line with the humanization hypothesis^19^, face photos may serve to humanize individuals, thereby encouraging empathy and understanding. Considering the current study, this could reduce the influence of negative reputations and facilitate more constructive dialogues and cooperative outcomes. In light of these findings, policymakers and organizations may consider applying these insights when designing interventions aimed at promoting collaboration and trust in various settings, including neighborhood initiatives, environmental conservation programs, or public health campaigns, where group cooperation is essential for success.

Regarding name identification, our findings suggest that names, if presented alone, are insufficient to increase cooperativeness, but when combined with reputation, they mitigate the reputation effect regardless of its direction. The diminishing negative effect of free-rider reputation is consistent with the (de)humanization literature, which shows that minimal information, like names, can attenuate the effects of egocentrism and abstract representations of others^35^. However, diminishing the effect of cooperative reputation does not follow the same logic, and some factors could explain this phenomenon. First, face processing is fast, automatic, and shaped by social information, which can modulate people’s social inferences and behavior^19^. On the other hand, names are usually generic, arbitrary, and could lead to associations with known people, potentially interfering with the processing of the graphs. Second, in the group responding to the name condition, there was a large presence of people with income between R$1,000 and R$2,000. In preliminary analysis (supplementary material), we observed that higher incomes increased the chances of being less cooperative. This question remains open to further investigations. We also chose neutral names from socioeconomic and racial perspectives. Therefore, we do not know if demographically stereotyped names, like the ones labeled as African-American “Lakisha” and “Jamal”^36^, or any other that could infer socioeconomic status, for example, could have led us to different results. Since we decided to minimize racial bias effects, future experiments should consider this manipulation.

A possible point of discussion is that in our study reputation information was related to the group of co-players rather than being individualized. As demonstrated by Schopler et al.^37^, group interactions tend to be more competitive than those between individuals. This could imply that we might expect lower cooperation in reputation groups if participants perceive the co-players as a "group," especially in neutral and free-rider conditions. However, our findings did not indicate any trends in this direction, as observed in the Name and Reputation group, where all conditions outcomes were similar to the control group.

As limitations, we did not control for facial characteristics such as facial expressions or racial traits (nose and lips shapes), since they were beyond the scope of the current study. Nonetheless, considering the existing literature about the effect of specific facial traits, if photos modulate reputation, the next step should be understanding how different facial characteristics may modulate distinct reputation levels. Furthermore, there might be differences between the exposure to face photos and photographs of eyes alone. We also acknowledge a potential limitation in our study related to the belief on the authenticity of the face photos and names used. Despite our intent, there's a possibility that some participants may not have believed that these elements were authentic. This could have influenced their perception of the interaction and, consequently, our results. The challenge of ascertaining participants' exact beliefs—even with explicit measures—remains an issue in this type of research. We suggest future researchers bear this consideration in mind and to explore strategies to mitigate its potential impact. Despite these limitations, our results suggest that the key to better understanding the effect of face unmasking in prosocial behavior may lie in investigating its modulatory aspect on reputation.

Overall, our research showed that, compared to the control group, reputation alone was relevant information—significantly increasing the participants’ cooperation during the cooperative condition (and displaying a statistical tendency in the neutral condition) and significantly decreasing cooperation during the free-rider condition. Additionally, it showed that identification alone (face or name) had no effect on the participants’ cooperativeness. However, when faces were combined with reputation, they significantly increased cooperation for neutral and cooperative conditions and mitigated the effect of free-rider reputations. Curiously, the positive effect of combining identification and reputation was specific to faces, as names with neutral or cooperative reputation showed no significant difference compared to the control group (except for a statistical tendency for the cooperative condition). However, similar to faces, names mitigated the effect of free-rider reputations and increased the positivity of neutral and cooperative reputations.

In our study, reputation itself is the most prevailing factor in determining cooperative behavior: free-rider conditions received fewer contributions than the cooperative ones. These results might seem contrary to Andreoni and Petrie’s^8^ findings because of their conclusion that the ‘information’ condition did not differ from the control regarding participants’ cooperative behavior. However, their methodological design differs from ours in some aspects, as previously mentioned. In our study, reputations were pre-defined, and all participants were exposed to free-riders, neutral, and cooperative co-players. Additionally, the fact that they conducted a real-time game provided participants with a different relationship with co-players: while our participants were only evaluating others’ reputations, in their study, participants were also building their own reputation. In the end, they found that the way co-players played influenced participants’ cooperative responses. Our study consolidates these conclusions by transforming different reputations into an experimental variable.

Our experiment seems aligned with a value-based perspective, which aims to understand how contextual factors and individual differences influence cooperation^38^. Considering that individuals’ social preferences shape outcome evaluations, including the decision to cooperate or defect, we studied how these two variables (reputation and identification) may modulate participants' responses. In general, we found evidence that some contextual variables that matter for cooperation interact hierarchically to modulate cooperative behaviors. Our results demonstrate that the decision to cooperate or not is built from a complex interaction between individual and contextual factors.

## Conclusion

Our study aimed to understand how unmasking co-players with face photos, names, and reputations could influence group-based cooperative decisions in a PGG. Furthermore, we also tried to comprehend if the identification could modify the evaluation of the reputational information. Considering our results in the three levels of reputational indexes we manipulated (neutral, free-rider, or cooperative), face photos modulated the impact of reputation on cooperation by (i) mitigating the negative impact on cooperation occurrence when facing a free-rider reputation and (ii) significantly increasing cooperation when the players appeared as neutral or cooperative. This modulation effect, however, was not extended to the other type of unmasking, the name condition. We found that the simple individualization of players by names is insufficient to modulate cooperation, at least when more complex information, like demographic stereotypes, is not embedded in these names. Together, our findings suggest a hierarchical interaction between reputation and identification as contextual information: reputation alone is enough to influence cooperation rates, and revealed identities modulate the weight of reputation on cooperative decision-making.

### Supplementary Information

Supplementary Information.

## Data Availability

Data and code for this paper can be found at the following link: https://osf.io/f7xn2/.
