# Supplementary material for: Effects of co-players' identity and reputation in the public goods game
Source: Sci Rep. 2023 Aug 19;13:13520. doi: 10.1038/s41598-023-40730-4 (PMC10439960; doi:10.1038/s41598-023-40730-4)
Supplement: Supplementary file 1 — Supplementary Information. [file 41598_2023_40730_MOESM1_ESM.docx]

**Supplementary Material**

**Effects of co-players' identity and reputation in the public goods game**

**Waldir M. Sampaio¹*, Ana Luísa Freitas¹, Gabriel G. Rêgo¹, Leticia Y. N. Morello¹, Paulo S. Boggio¹*.**

¹Social and Cognitive Neuroscience Laboratory, Mackenzie Presbyterian University, São Paulo, Brazil.

***Corresponding Authors:**

Waldir M. Sampaio, Social and Cognitive Neuroscience Laboratory, Mackenzie Presbyterian University, Rua Piauí, 181, 10th Floor, São Paulo 01241-001, Brazil. E-mail: waldirsampaio1994@gmail.com

Paulo S. Boggio, Social and Cognitive Neuroscience Laboratory, Mackenzie Presbyterian University, Rua Piauí, 181, 10th Floor, São Paulo 01241-001, Brazil. E-mail: paulo.boggio@mackenzie.br

Section 1. Cooperation occurrence along the trials

[Section 2. Face photos selection, validation, and manipulation](#_48bddch8svs1)

[Section 3. Names selection](#_k8cxc6rerds)

[Section 4. Verbatim of the Public Goods Game instructions](#_vqi0na59wf37)

[References](#_mlg509lbg3k1)

**Section 1. Cooperation occurrence along the trials per group**

Figure S1. Cooperation occurrence along the trials per group
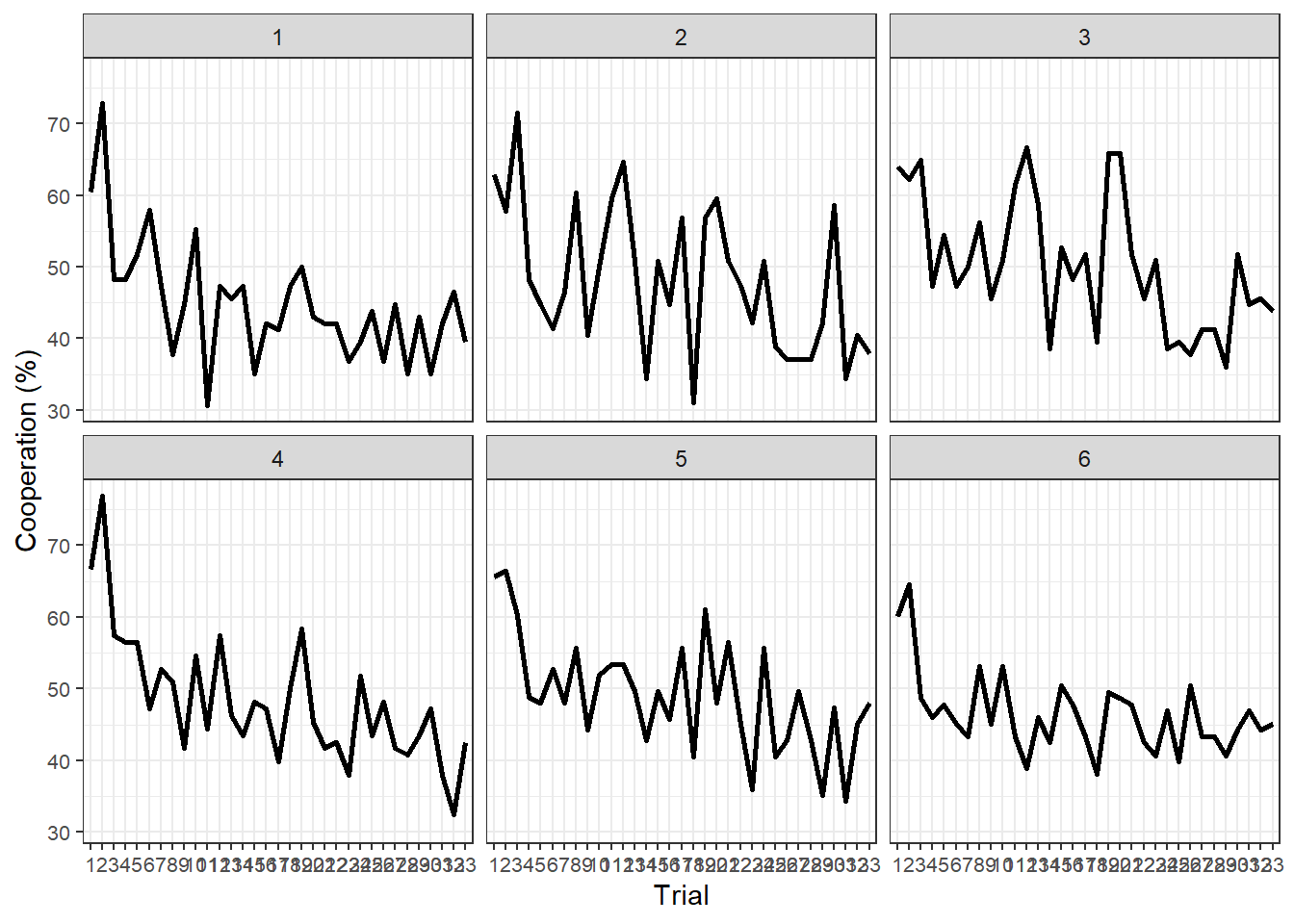


*Note.* Proportion of cooperation occurrence in each trial in every group. 1) Control; 2) Reputation; 3) Face ID; 4) Face ID and Reputation; 5) Name ID; 6) Name ID and Reputation.

##

## **Section 2. Face photos selection, validation, and manipulation**

Each photo was evaluated by at least 20 evaluators who were not involved in the study. These evaluators classified the person in the photo into one of the following categories: 1) White; 2) Black; 3) Mixed-race; 4) Asian; 5) Native Brazilian Indigenous. Only images that were considered as “white” by at least 95% of the evaluators were included in the study.

After this process, we used FaceAPP (2022) to apply a filter to the selected images, making their facial expressions more neutral. Image ‘A’ is the original photo before the application of the filter, while image ‘B’ shows the photo after the filter has been applied.

1. **B)**


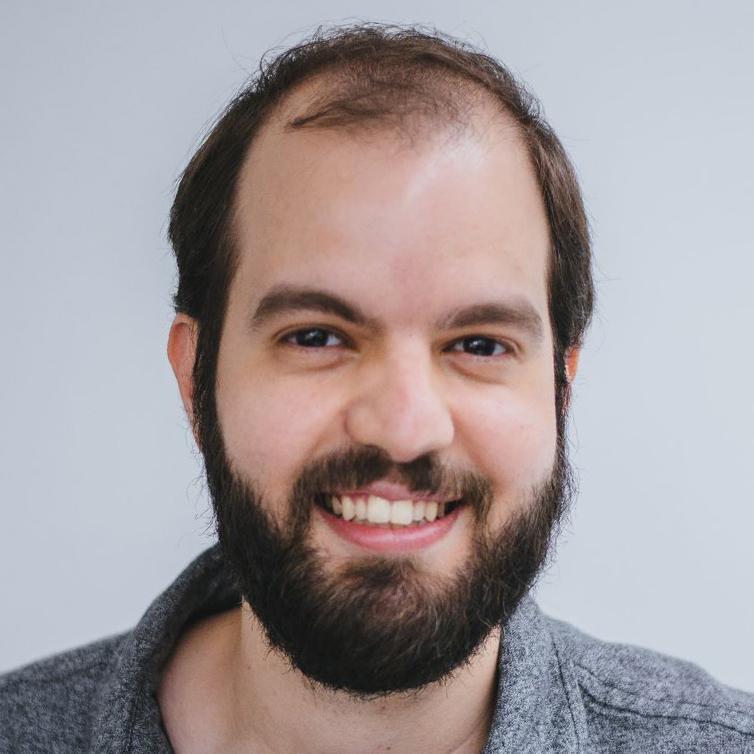

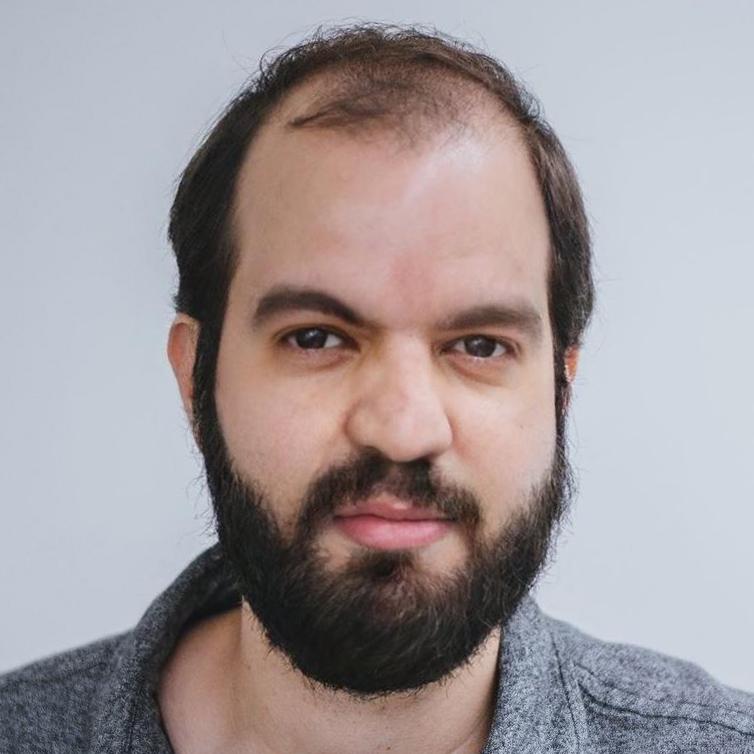


Note: Due to copyright issues, the images used in the experiment screens have been replaced in the supplementary material with photographs of real people who authorized the use of their images for the publication of this study. In the images A and B, the selected photograph underwent the same procedure of facial expression manipulation that was applied to the images from the software 'This person does not exist'.

##

## **Section 3. Names selection**

As stated in the article, we selected 99 names (3 names per round, for a total of 33 rounds) from the list of the 100 most registered birth names in Brazil in 2018 (Cerioni, 2018). The selected names, the rounds in which they were used, and their respective players' positions were:

**Table S3**

*Selected names and their positions in the experiment trials*

| Trial | 1st Player | 2nd Player | 3rd Player |
| --- | --- | --- | --- |
| Trial 1 | Miguel | Arthur | Heitor |
| Trial 2 | Helena | Alice | Manuela |
| Trial 3 | Bernardo | Valentina | Davi |
| Trial 4 | Sophia | Théo | Isabella |
| Trial 5 | Heloísa | Lorenzo | Gabriel |
| Trial 6 | Luísa | Pedro | Júlia |
| Trial 7 | Benjamin | Lorena | Matheus |
| Trial 8 | Livia | Lucas | Maria Clara |
| Trial 9 | Nícolas | Cecilia | Joaquim |
| Trial 10 | Eloá | Samuel | Giovana |
| Trial 11 | Henrique | Maria Clara | Rafael |
| Trial 12 | Maria Eduarda | Guilherme | Enzo |
| Trial 13 | Murilo | Benicio | Gustavo |
| Trial 14 | Isaac | João Miguel | Mariana |
| Trial 15 | Lara | Beatriz | Antonella |
| Trial 16 | Maria Júlia | Bento | Emanuelly |
| Trial 17 | João Miguel | Isadora | Lucca |
| Trial 18 | Ana Clara | Enzo Gabriel | Melissa |
| Trial 19 | Pedro Henrique | Ana Luiza | Felipe |
| Trial 20 | Ana Júlia | João Pedro | Esther |
| Trial 21 | Pietro | Lavínia | Anthony |
| Trial 22 | Maitê | Daniel | Maria Cecilia |
| Trial 23 | Bryan | Maria Alice | Davi Lucca |
| Trial 24 | Sarah | Leonardo | Elisa |
| Trial 25 | Vicente | Liz | Eduardo |
| Trial 26 | Yasmin | Gael | Isabelly |
| Trial 27 | Antônio | Alicia | Vitor |
| Trial 28 | Catarina | Gabriela | Vinicius |
| Trial 29 | Clara | Noah | Isis |
| Trial 30 | Caio | Rebeca | João |
| Trial 31 | Rafaela | Emanuel | Marina |
| Trial 32 | Cauã | Ana Laura | João Lucas |
| Trial 33 | Maria Helena | Calebe | Agatha |

##

## **Section 4. Verbatim Translation of the Public Goods Game instructions**

Here, we present a literal translation of the instructions given to the participants for the Public Goods Game. Each translated screen is followed by the original screen in Brazilian Portuguese. Furthermore, on screens where additional explanation is necessary, our commentary is bolded and enclosed within square brackets.

**Screen 1.** Please read the following instructions carefully.

You will participate in a series of interactions. In each round, you will interact with three other people. Each interaction will consist of a totally different group of people. In other words, during the study, you will NEVER interact with the same person more than once.

Please press SPACEBAR to continue.


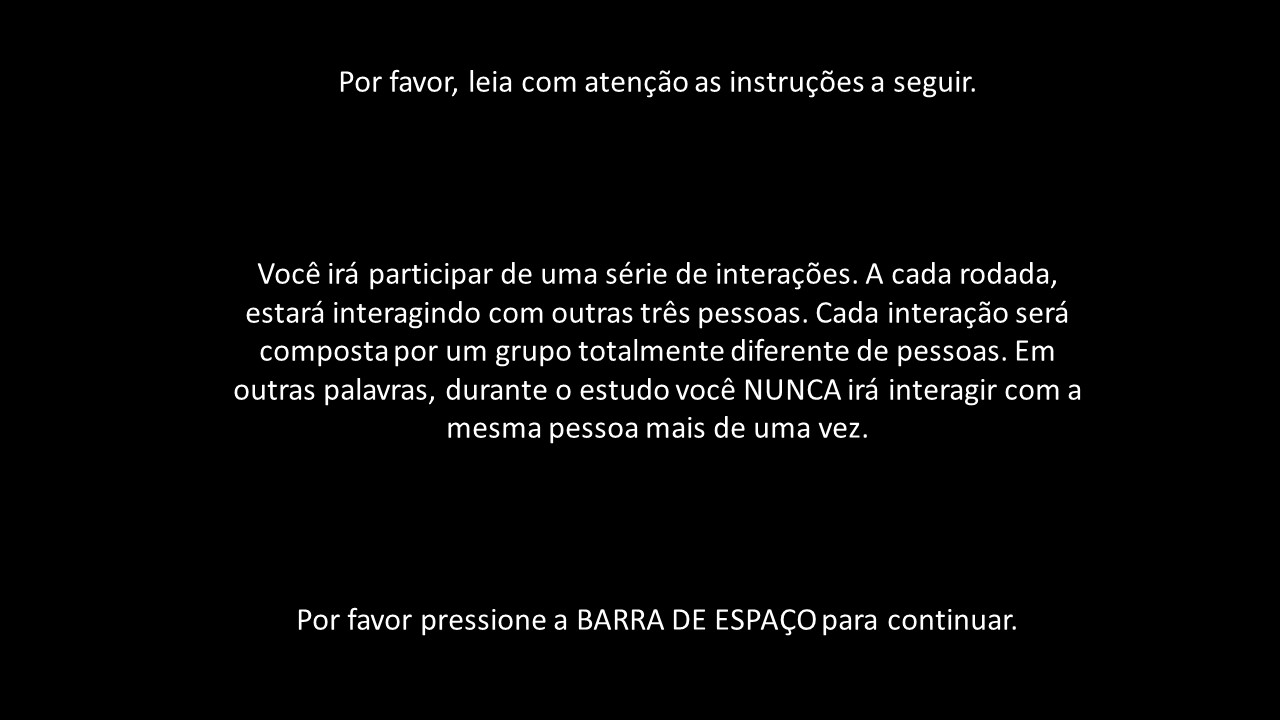


**Screen 2.** These interactions will not happen in real time. Instead, you will play with other participants’ responses, that were recorded in previous studies. Likewise, your responses will be anonymously recorded and used in future studies.

In other words, the decisions you make today will affect both yours and future participants’ monetary distributions.

Please press SPACEBAR to continue.


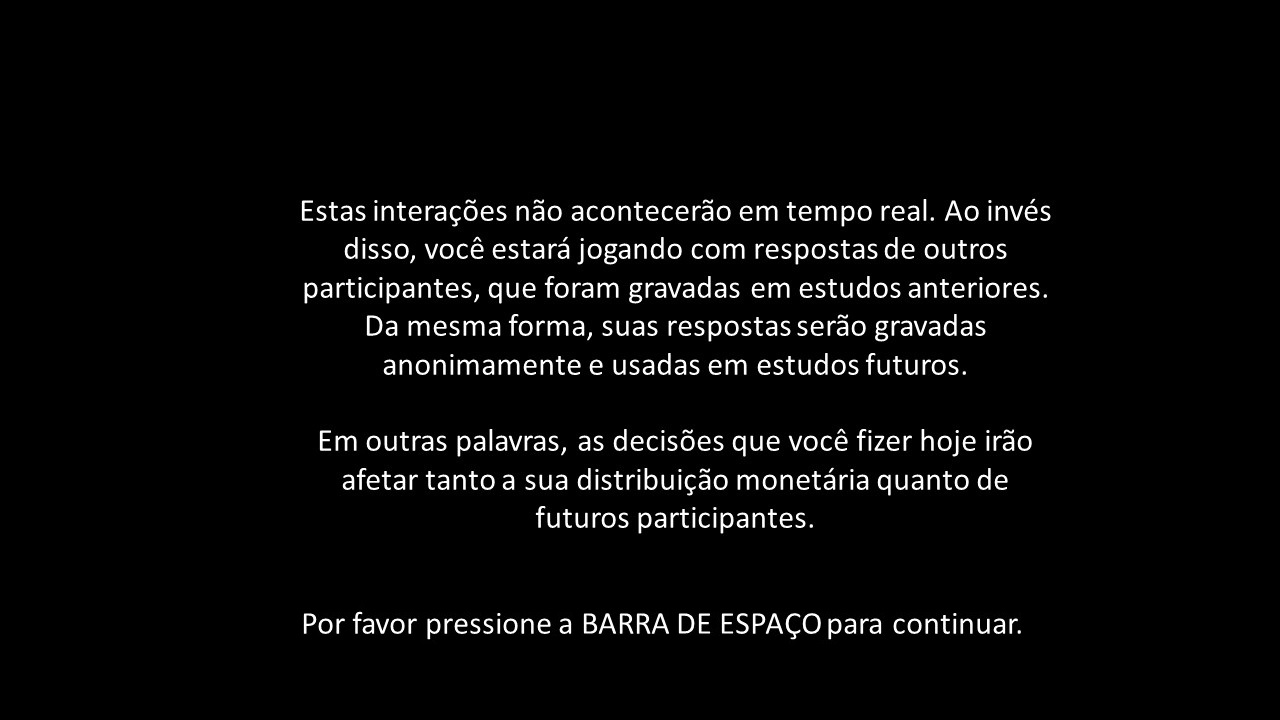


**Screen 3.** During each interaction, each player will receive R$8 and must choose one of the following decisions:

Each player could keep R$8 for him/herself

OR

Each player could give R$8 to the group

Please press SPACEBAR to continue.


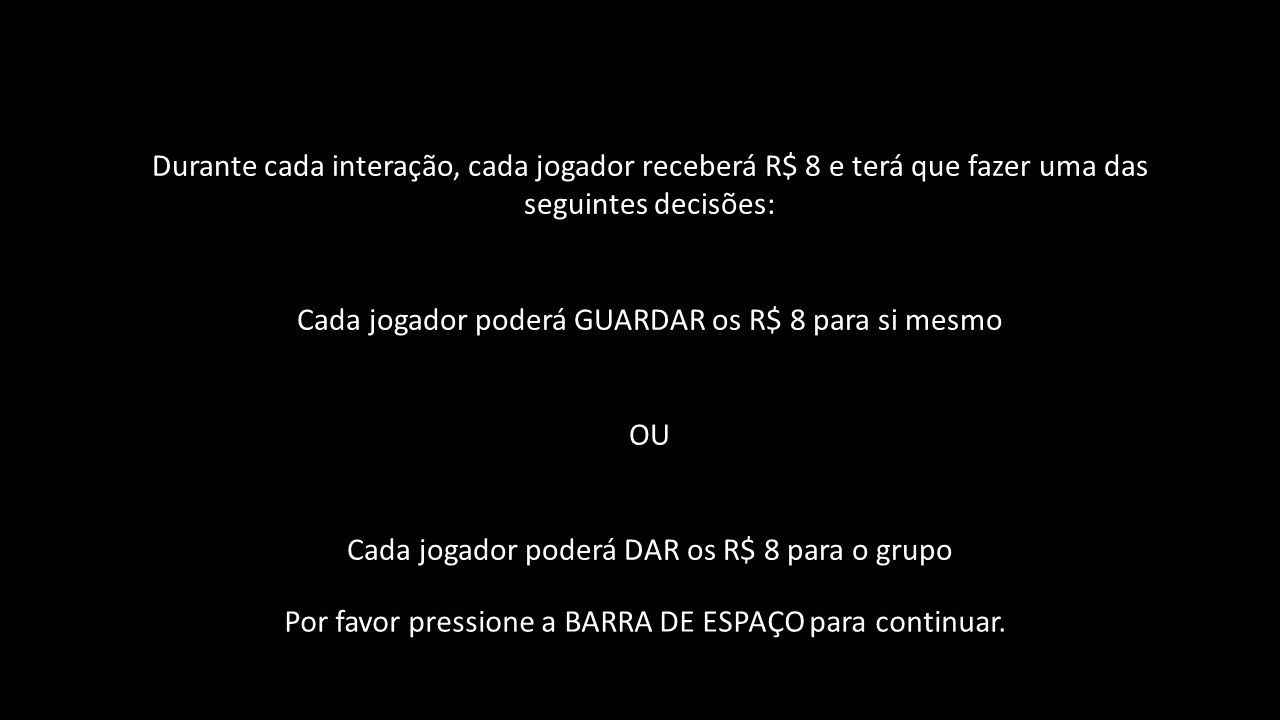


**Screen 4.** It is important to know that, in each round, the computer will multiply by 2 the whole given amount and will equally share the outcome among the four players.

The amount each player will receive in each round depends on the choices each player will make, whether or not to give the money to the group.

Please press SPACEBAR to continue.


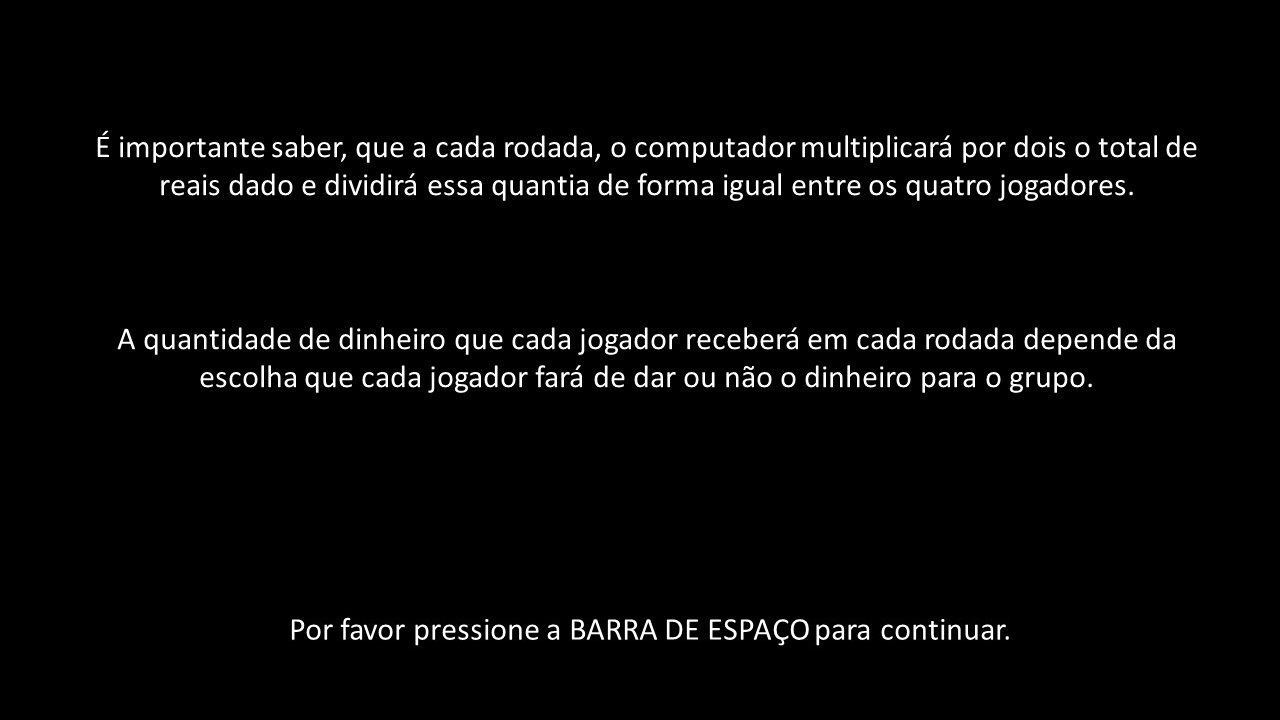


**Screen 5.** At the beginning of each round, the words NEW PLAY will appear to notify you of the start of each turn. Then the SAVE or GIVE buttons will appear on the screen and you will receive R$8, in the middle of the screen, as shown in the example below.

Please press SPACEBAR to continue.

**
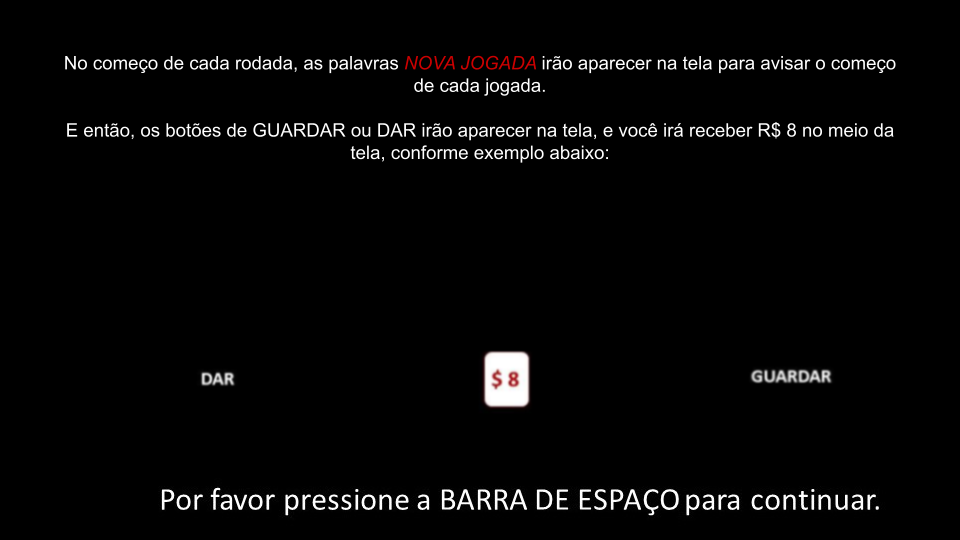
**

**[This screen presents differences for each experimental group. The one shown above corresponds to the control group. In the other groups, in addition to the information already described, information about the specific experimental variable was presented. Below, we present the description and the extra image that was inserted for example purposes.]**

**Face ID Group.** Simultaneously, a photo of the players participating in the round will be shown. In the experiment, these face photos were generated by AI. In this supplementary material, the photos are of real people who have consented to the use of their images for the publication of this study.


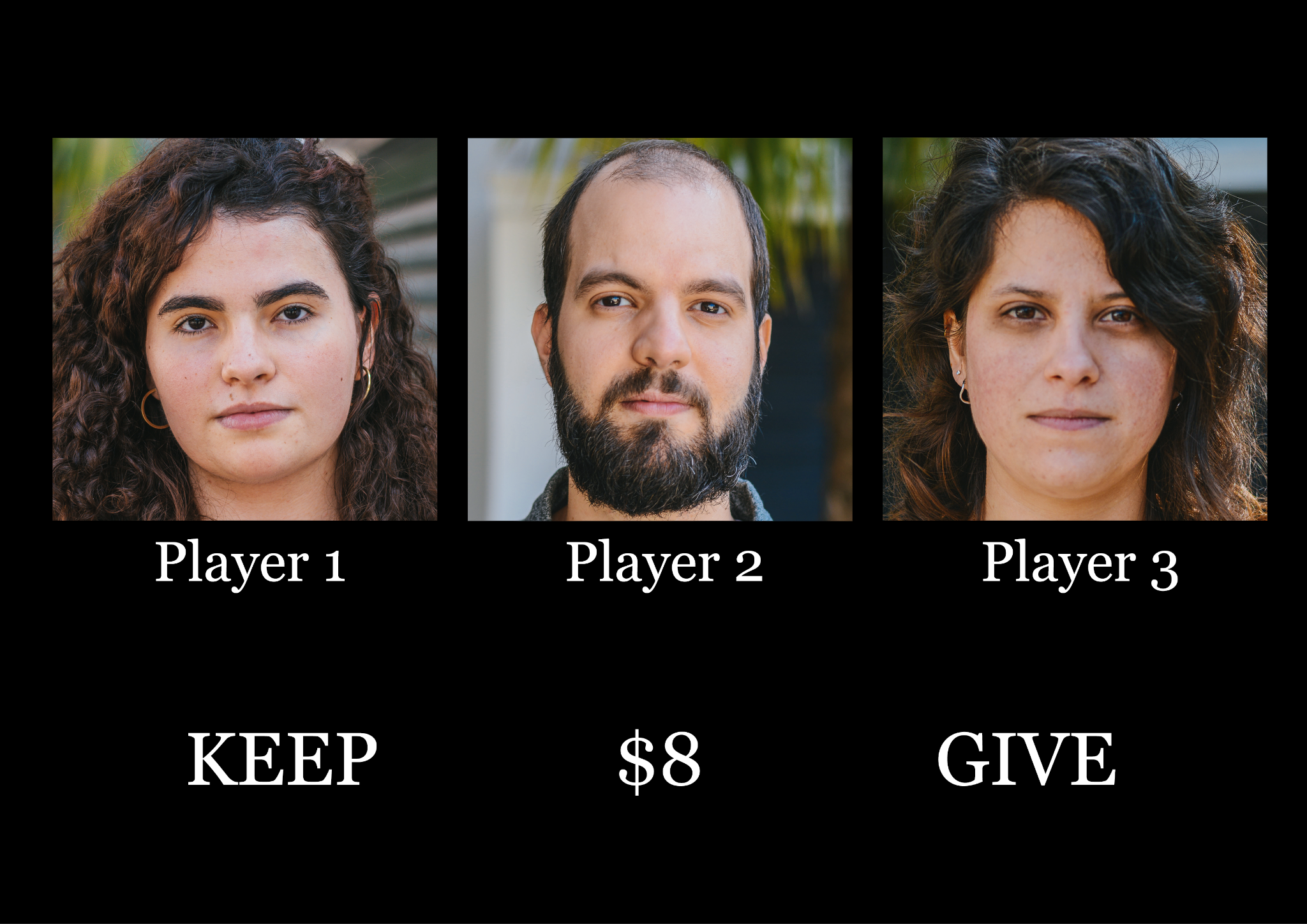


**Reputation Group.** At the same time, a bar graph containing information on how many times the group has fully cooperated in the last 20 rounds will be shown.


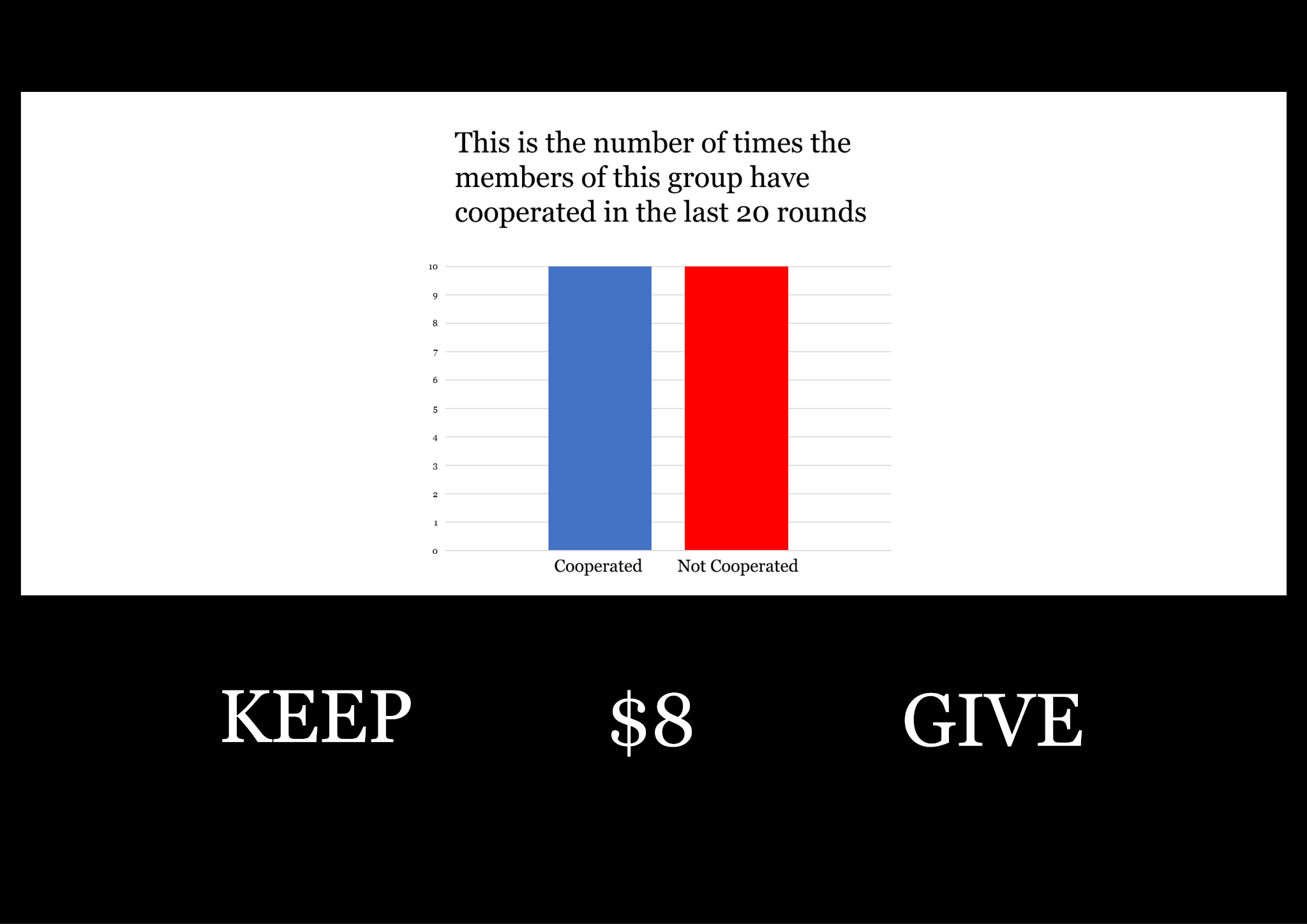


**Reputation and Face ID Group.** At the same time, a photo of the players who will participate in the round will be shown with a bar graph containing information on how many times the group has fully cooperated in the last 20 rounds. In the experiment, these face photos were generated by AI. In this supplementary material, the photos are of real people who have consented to the use of their images for the publication of this study.


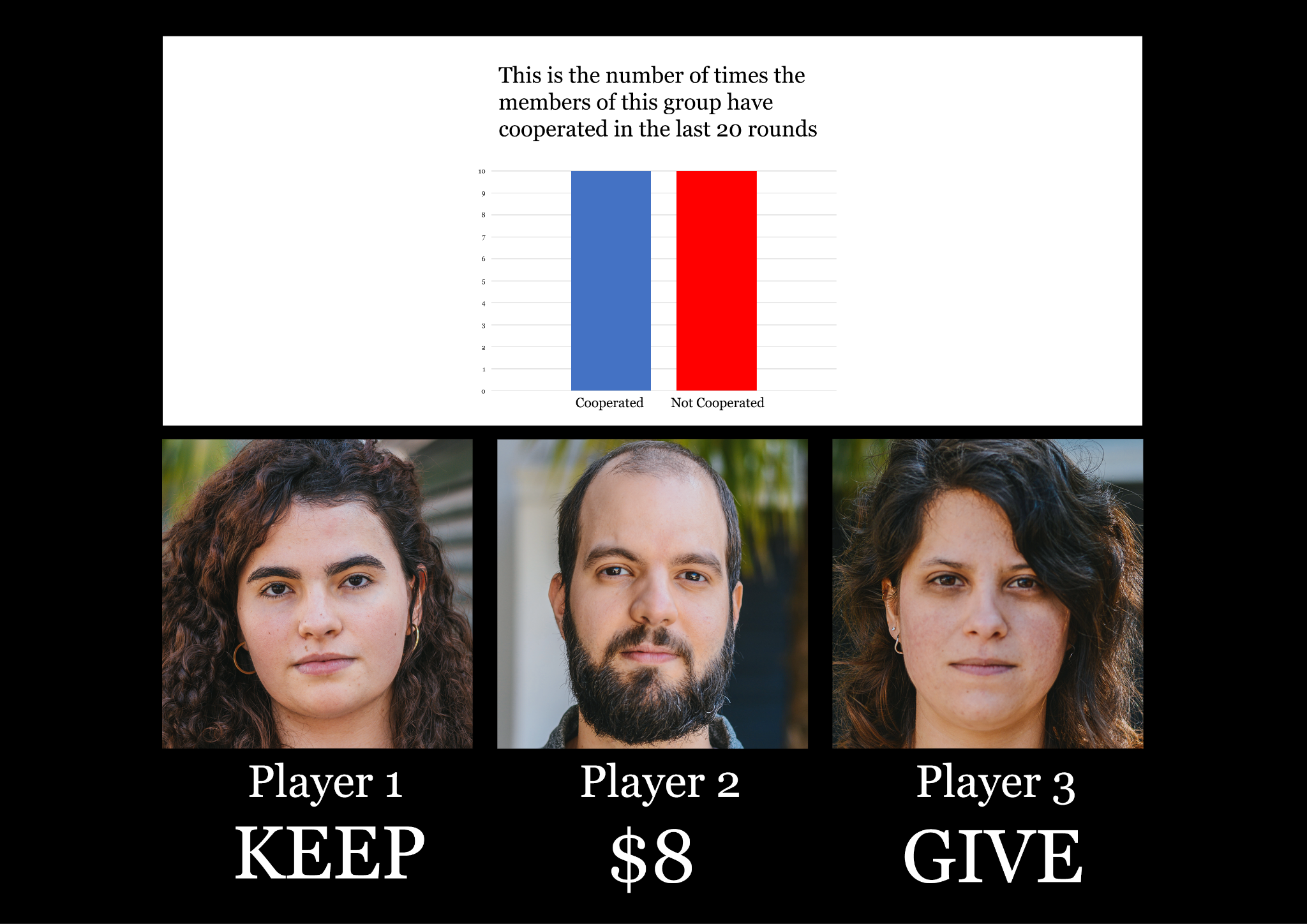


**Name ID.** At the same time, the names of the participants from that round will be shown.


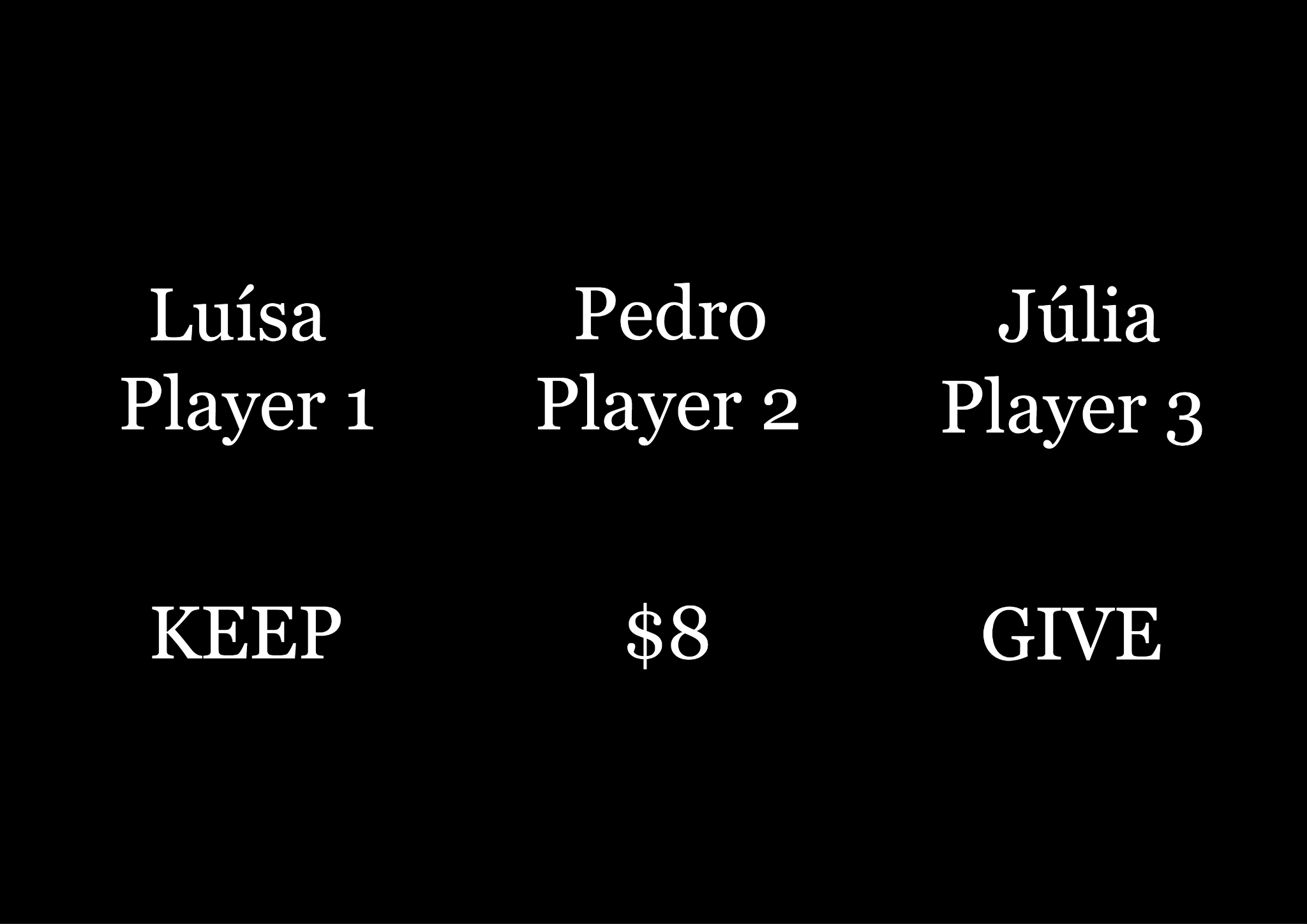


**Reputation and Name ID Group.** At the same time, a bar graph containing information on how many times the group has fully cooperated in the last 20 rounds will be shown with the names of the participants from that round.


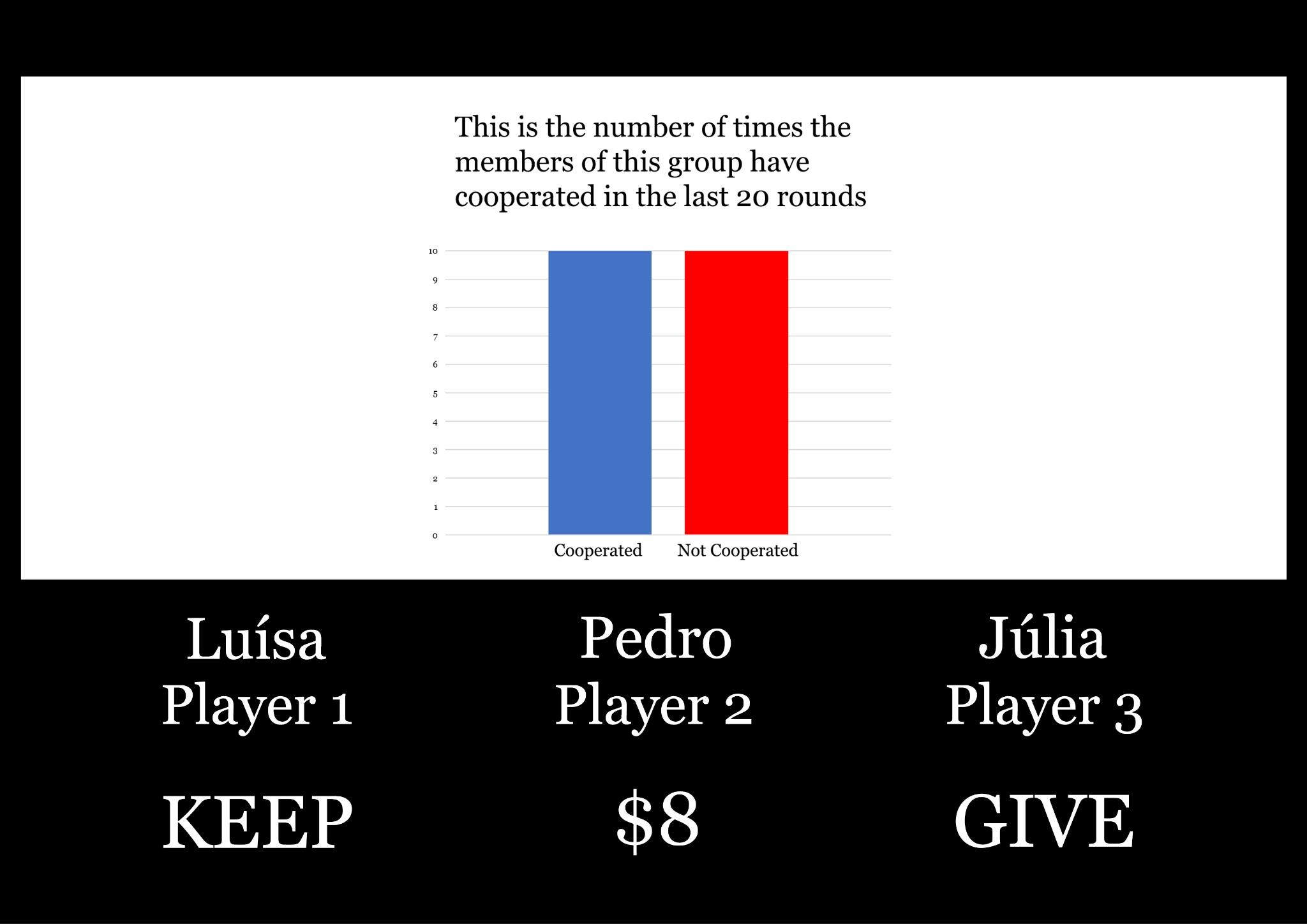


**Screen 6.** Your job is to choose whether to SAVE or GIVE the money to the group by pressing:

Mouse left click on GIVE or SAVE

GIVE R$8 KEEP

Please press SPACEBAR to continue.


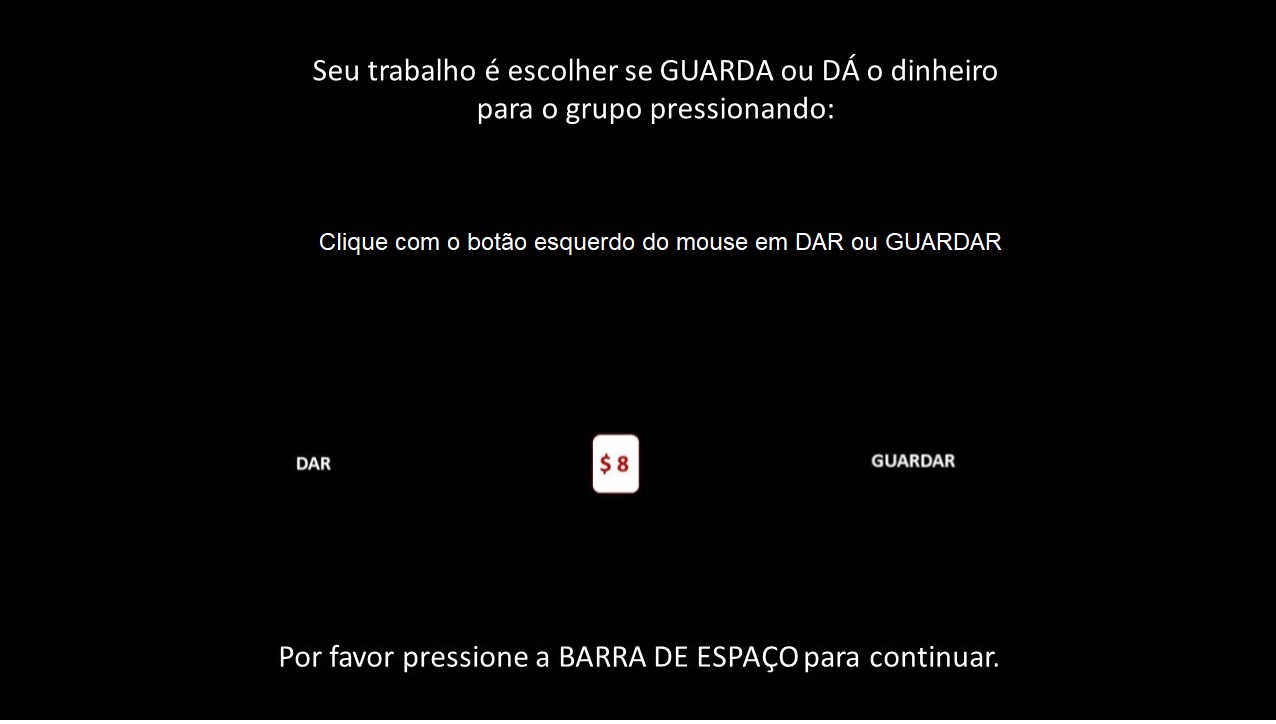


**Screen 7.** After all participants made their decisions, four figures will appear, revealing the decision of each player and the outcome of their payout as well.

If the player's figure is BLUE, it means the player decided to GIVE his/her money to the group.

If the player's figure is YELLOW, it means the player decided to KEEP his/her money for him/herself.


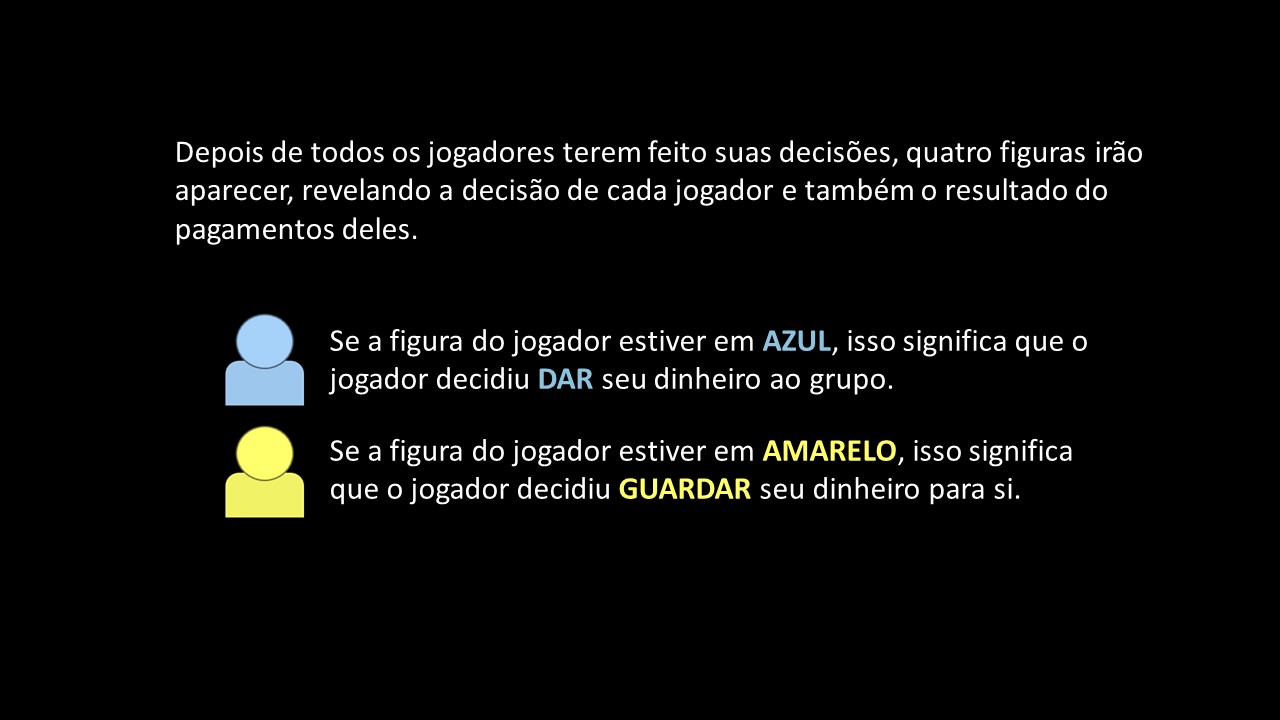


**Screen 8.** For example, the following return means that you, Player 1, and Player 3 decided to GIVE your own R$8 (each) to the group, while Player 2 decided to KEEP his/her R$8 for him/herself.

SAMPLE PICTURE

We will work through your payment next.


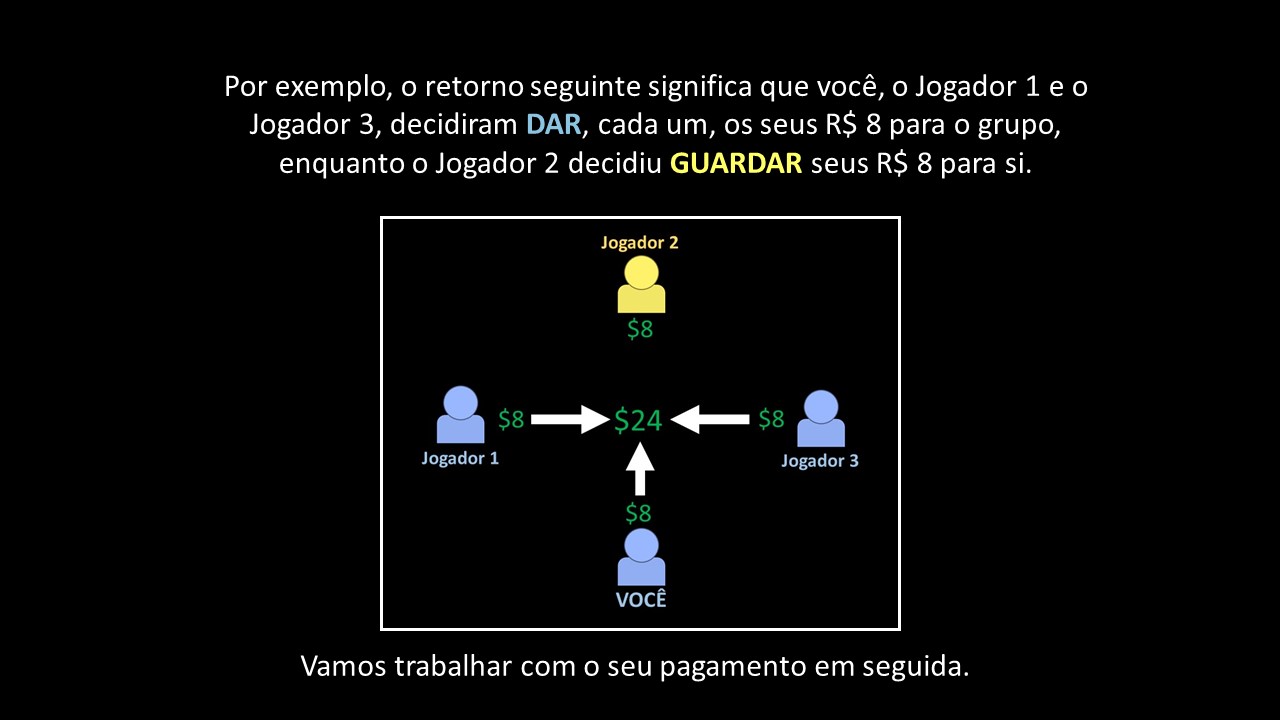


**Screen 9.** If you GAVE your R$8 to the group, they would be added to the R$8 that Player 1 and Player 3 gave (R$8 + R$8 + R$8 = R$24),

multiplied by 2 (2*R$24=R$48),

and then equally divided among the group (R$48/4=R$12)

You would get R$12 in total.

**
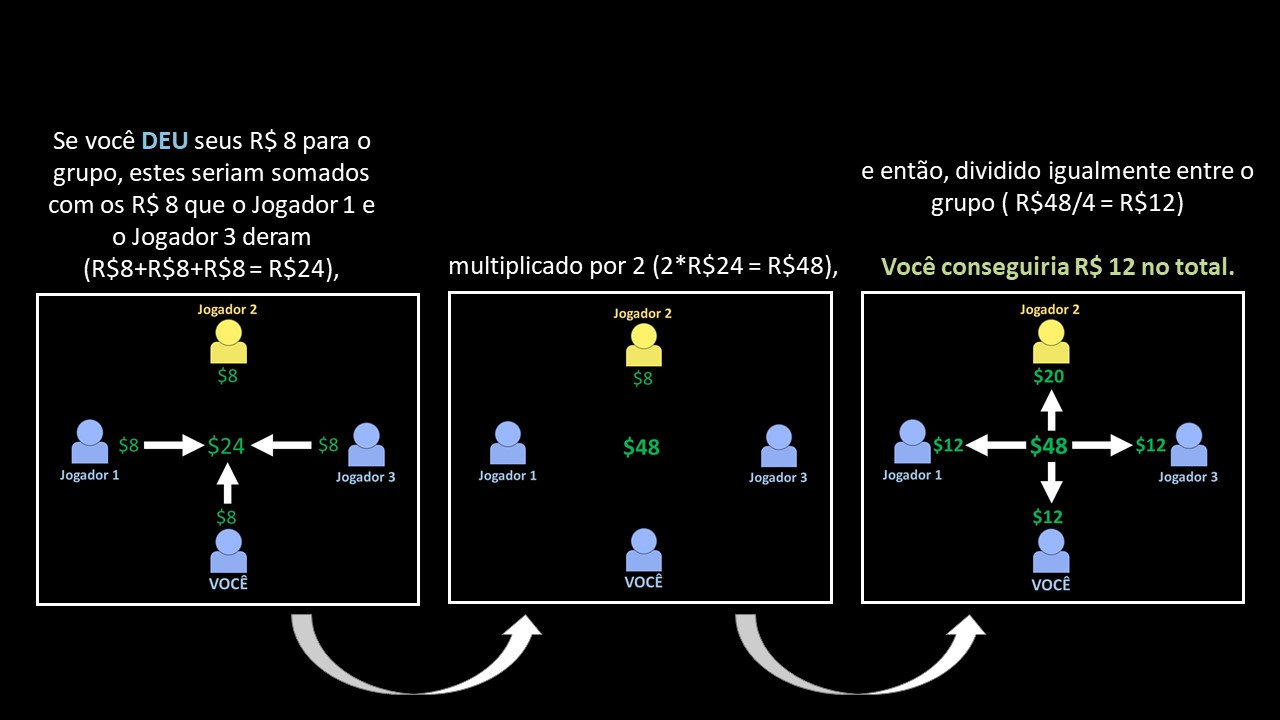
**

**Screen 10.** If you SAVED your R$8 for yourself, the R$8 that Player 1 and Player 3 gave to the group would be added up (R$8+R$8=16),

multiplied by 2 (2*R$16=R$32),

and then equally shared among the group (R$32/4=R$8). So, added to the R$8 you saved, you would have an additional of R$8 (R$8+R$=R$16)

You would get R$16 in total.


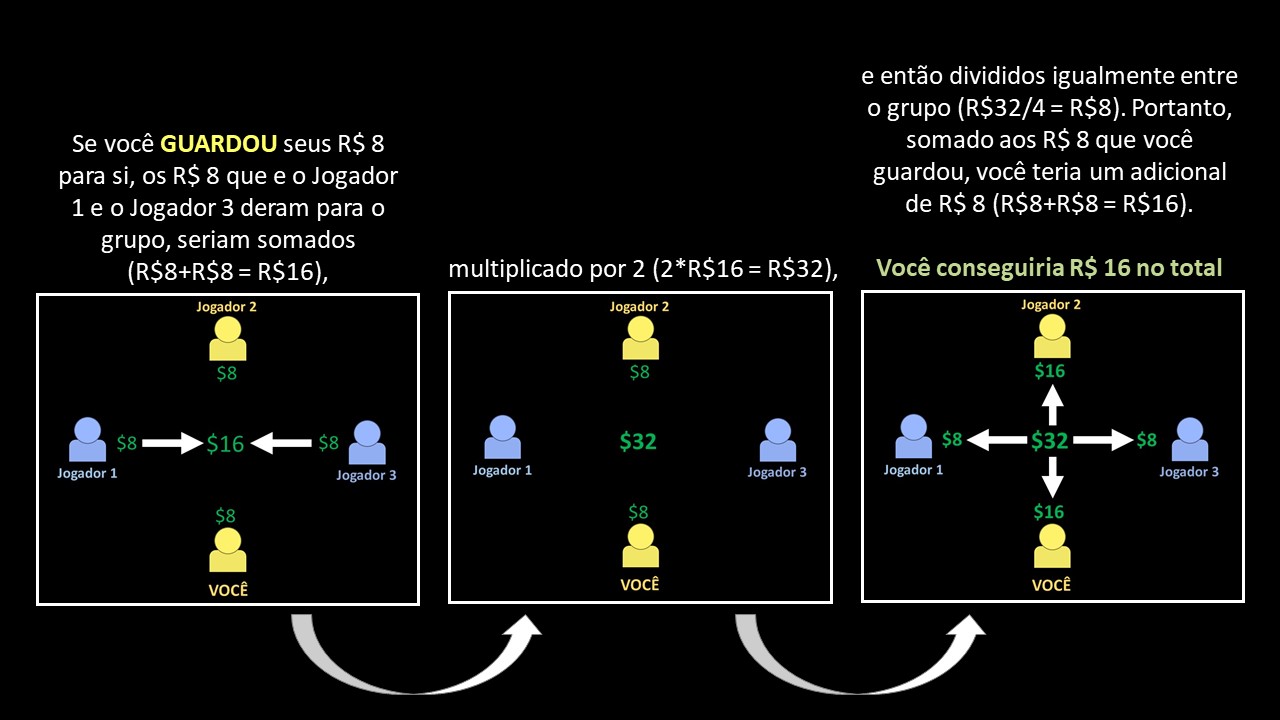


**Screen 11.** Finally, after you received all the feedback about what happened in the previous turns, the words NEW PLAY will appear to indicate that you are ready for the next turn.

Right after, the answering buttons will appear and you will receive R$8 in the middle of the screen. Again, your job is to choose between pressing SAVE or GIVE this money to the group.

Once you have decided, the process will restart in the next round.


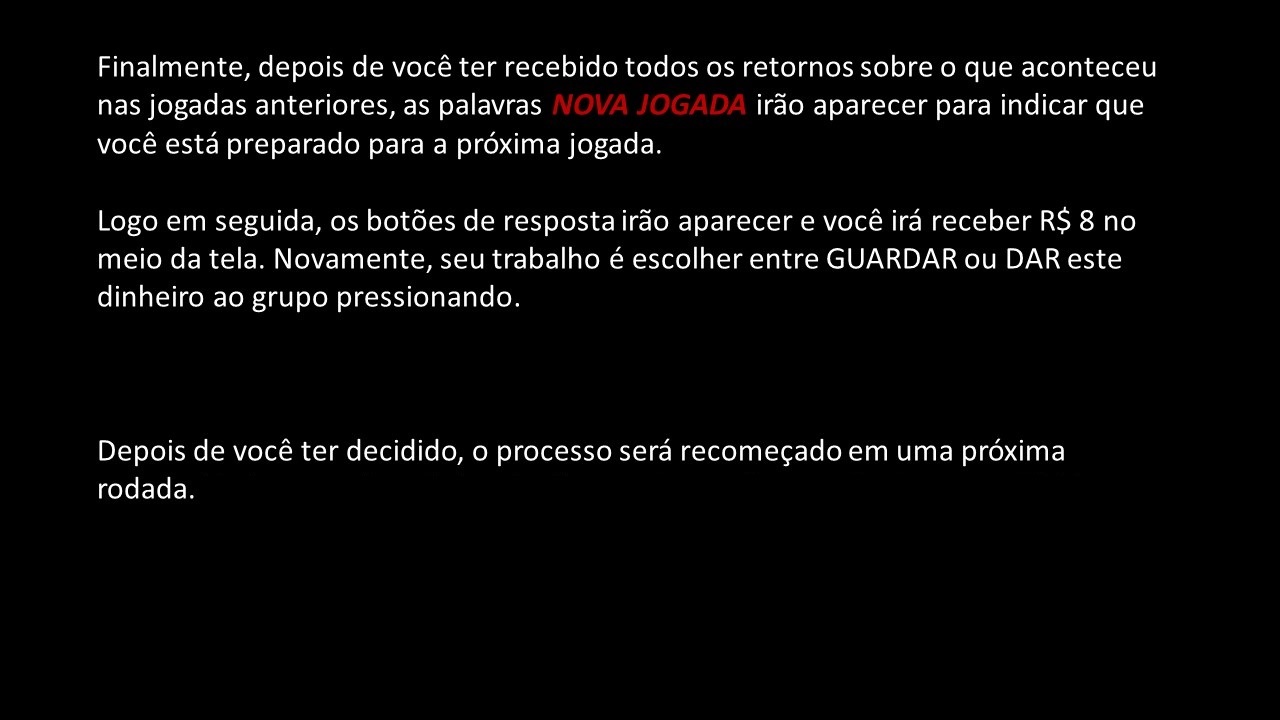


## **References**

FaceApp. (2022). FaceAPP: Editor facial ideal (version: 10.1.0) [Mobile app]. Apple AppStore. <https://apps.apple.com/br/app/faceapp-editor-facial-com-ia/id1180884341>

Cerioni, C. (2018, November 29). Os 100 nomes de bebês brasileiros mais populares em 2018.Exame. https://exame.com/brasil/os-100-nomes-de-bebes-brasileiros-mais-populares-em-2018/
